# Application of Optimal Control Theory to Fourier Transform Ion Cyclotron Resonance

**DOI:** 10.3390/molecules26102860

**Published:** 2021-05-12

**Authors:** Vardan Martikyan, Camille Beluffi, Steffen J. Glaser, Marc-André Delsuc, Dominique Sugny

**Affiliations:** 1 Laboratoire Interdisciplinaire Carnot de Bourgogne (ICB), UMR 6303 CNRS-Université Bourgogne-Franche Comté, 9 Av. A. Savary, BP 47 870, F-21078 Dijon, France; martikyan.vartan@yandex.com; 2 CASC4DE S.A.S, Pole API Batiment 1, 300 Boulevard Sébastien Brant, 67400 Illkirch, France; camille.beluffi@casc4de.eu (C.B.); delsuc@igbmc.fr (M.-A.D.); 3 Department of Chemistry, Technische Universität München, Lichtenbergstrasse 4, D-85747 Garching, Germany; glaser@tum.de; 4 Munich Center for Quantum Science and Technology (MCQST), Schellingstrasse 4, 80799 München, Germany; 5 IGBMC, 1 rue laurent Fries, BP 10142, 67404 Illkirch, France

**Keywords:** optimal control, robust protocol, Ion Cyclotron Resonance

## Abstract

We study the application of Optimal Control Theory to Ion Cyclotron Resonance. We test the validity and the efficiency of this approach for the robust excitation of an ensemble of ions with a wide range of cyclotron frequencies. Optimal analytical solutions are derived in the case without any pulse constraint. A gradient-based numerical optimization algorithm is proposed to take into account limitation in the control intensity. The efficiency of optimal pulses is investigated as a function of control time, maximum amplitude and range of excited frequencies. A comparison with adiabatic and SWIFT pulses is done. On the basis of recent results in Nuclear Magnetic Resonance, this study highlights the potential usefulness of optimal control in Ion Cyclotron Resonance.

## 1. Introduction

Performing efficient and robust state control by means of external time-dependent system parameter is a fundamental challenge in many technological developments at macroscopic or microscopic scale [1,2,3,4]. In this latter case, open-loop control protocol, i.e., without any real time feedback from the experiment during the control process, is generally used for practical and technical reasons. The controls are only designed from a modeling of the system dynamics and the efficiency of the control scenario may suffer from the accuracy of the theoretical description. The robustness of a control process with respect to experimental imperfections is therefore a key parameter in view of experimental implementation. Different techniques extending from adiabatic pulses to optimal control theory (OCT) have been developed in this open-loop framework to find the pulse parameters [1,3,5,6,7,8]. Optimal control tackles the question of bringing a dynamical system from one state to another with minimum expenditure of time and resources [1,2,3,4]. The modern version of OCT was born in the 1960s with the Pontryagin Maximum Principle (PMP), which provides a general and rigorous mathematical framework for optimal control techniques [9,10,11,12,13]. OCT has become nowadays a key tool in many different domains extending from space dynamics to robotics or quantum mechanics [1,3,11]. Optimal process is defined from a cost functional (to minimize) which can depend on the state of the system and the control field. For systems with complex dynamics and optimization targets which are difficult to reach, it is necessary to use optimal control algorithms converging iteratively towards the optimal solution. The flexibility of this approach makes it possible to adapt this tool to any experimental situation. Generally, it is possible to include constraints in the algorithms to account for requirements related to a specific material or device [1,3]. The only relative limitation concerns the accuracy of the modeling, even if robustness can be improved by controlling simultaneously an ensemble of systems which differ by the values of one or several constant parameters [14,15,16,17]. OCT has been applied to quantum systems first in the context of physical chemistry to steer chemical reactions or control specific degrees of freedom [5,18], followed by control of spin dynamics [19,20] for applications in Nuclear Magnetic Resonance (NMR) [15,17,21,22,23,24,25] and Magnetic Resonance Imaging [26,27,28,29]. It has become a key tool in this domain to improve the efficiency and the sensitivity of standard experimental setups [3]. In NMR and quantum physics, a well-known optimization method is GRAPE [30], which is a gradient-based algorithm [1]. This approach has been applied with success in many different contexts.

Fourier-Transform Ion Cyclotron Resonance (ICR) Mass Spectrometry [31,32] is a type of mass spectrometry based on cyclotron frequency of ions in a fixed magnetic field [33,34,35]. Ions are excited at their resonant cyclotron frequencies to larger cyclotron radii by an electric field orthogonal to the magnetic field. After the excitation pulse, the ions rotate freely with a frequency characteristic of their mass. The image current induced by the ions on a pair of electrodes is detected. The Fourier transform of the resulting transient signal leads to the mass spectrum after a proper calibration. In a homogeneous magnetic field, ICR allows accessing the highest resolution available in mass spectrometry, while leading to extreme sensitivities. This spectrometry has experienced a recent renewal based on several methodological improvements and the search for very high resolutions, which are required to study complex biological or environmental mixtures. Several techniques developed and used in ICR have been inspired by equivalent approaches in NMR. An example is given by two-dimensional ICR [36,37,38,39,40,41] which was proposed in analogy to two-dimensional NMR spectroscopy [19,20]. Following this fruitful approach and given the success and efficiency of optimal control techniques in NMR, a question which naturally arises is the application of this method in ICR. This paper aims at taking a step toward the answer to this open issue. ICR Mass Spectrometry can provide very high resolution mass spectra over a large range of mass to charge ratio. In the ICR experiment, ions are initially at rest in the center of the trap, and they have to be excited to generate a resonant signal which can cover, in broad band experiments, frequencies from a few 10 s kHz for high m/z up to 1 MHz or higher for the fastest species. However, this implies that all ions have to be excited over this frequency range in an even and controlled manner.

We explore in this study how optimal control can be used to design efficient and robust excitation pulses in ICR. To the best of our knowledge, this has never been studied. Due to the wide bandwidth of ICR signal, excitation pulses are usually simple *chirped* adiabatic pulses with a frequency sweep. Some variations have been proposed such as off-resonance monochromatic pulses for selective excitation of given ions. Based on the linearity of ion dynamics, it has also been proposed to generate pulses by Fourier synthesis from a given excitation profile, in an approach called SWIFT (for Stored-waveform Inverse Fourier Transform) [42,43,44]. Optimal control is expected to allow a much wider range of possibilities such as the control of trajectory for given initial and final positions of the ion packet and for a given range of frequencies. To evaluate the contribution of OCT in ICR, we consider in this study the simplest modeling which is experimentally relevant. The experiment is considered in a simplified environment, with a constant magnetic field and a time-dependent homogeneous electric field oriented along a single axis orthogonal to the magnetic field and with no static component. This geometry is unrealistic, as there is no trapping potential, but allows to consider the dynamics of the ions restricted to a plane with a pure cyclotron trajectory and a zero magnetron component. The time-dependent electric field aims at exciting in a robust manner an ensemble of different ions from the center of the cell to a final position which depends in a controlled way of the ion frequency. The linearity of ion dynamics simplifies drastically the derivation of the optimal control law [10,45,46]. If there is no constraint on the intensity of the electric field, linear quadratic optimal control theory (LQOCT) can be applied. Many mathematical results have been established in this case [10,47] and the optimal solution can be derived analytically. When constraints are accounted for, a numerical algorithm has to be used to solve the optimal equations. Note that very few studies have solved optimal control problems of linear systems at the microscopic scale [47,48,49,50,51]. ICR is an interesting example, relevant experimentally, to stimulate further work in this direction.

The remainder of this paper is organized as follows. The formulation of the control problem and the description of the model system are outlined in Section 2. After a brief introduction to the principles of OCT, we apply OCT to ICR in Section 3. We describe the optimal control algorithm which allows taking into account experimental constraints on the control field. Numerical results in different experimental situations are given in Section 4. A comparison is made with the adiabatic and the SWIFT approaches. We conclude in Section 5 with an outlook and future perspectives. The Rotating Wave Approximation (RWA) is discussed in Appendix A. Technical details about the adiabatic and SWIFT techniques are presented, respectively, in Appendix B and Appendix C. The application of LQOCT is described in Appendix D.

## 2. Formulation of the Control Problem

### 2.1. The Model System

We consider the simplest modeling of ion trajectories in ICR. The different ions in the experimental cell are confined in the (x,y)-plane and are subjected to a constant magnetic field $\vec{B}$ and a time-dependent electric field $\vec{E}$, respectively, along the *z*- and *x*-axes of the laboratory frame. Note that optimal control techniques can also be used if two control fields along the *x*- and *y*-directions are available. The dynamics are governed by the Lorentz’s equation:

(1) $$m_{k}{\dot{\vec{v}}}_{k}=q_{k}\vec{E}+q_{k}\left({\vec{v}}_{k}\times \vec{B}\right),$$

where $m_{k}$, $q_{k}$ and ${\vec{v}}_{k}$ are the mass, charge and speed of the ion *k*. ${\dot{\vec{v}}}_{k}$ denotes the time derivative of ${\vec{v}}_{k}$. Equation (1) can be expressed as:

(2) $$\left\{\begin{array}{c} {\dot{x}}_{k}=v_{x_{k}}\\ {\dot{y}}_{k}=v_{y_{k}}\\ {\dot{v}}_{x_{k}}=\omega_{k}\left(e_{x}+v_{y_{k}}\right)\\ {\dot{v}}_{y_{k}}=-\omega_{k}v_{x_{k}}. \end{array}\right.$$

with the cyclotron frequency $\omega_{k}=\frac{q_{k}B}{m_{k}}$ and $\vec{e}=\vec{E}/B$. The coordinates $\left(x_{k},y_{k}\right)$ and $\left(v_{x_{k}},v_{y_{k}}\right)$ describe, respectively, the position and the speed of the ion *k* in the (x,y)-plane. We assume that the frequency $\omega_{k}$ belongs to the interval $\left[\omega_{min},\omega_{max}\right]$, which is defined by the ion packet under study. As described below, the aim of the control process is to excite the different ions in a robust way with respect to the parameter ω.

The control problem can be defined as follows. Starting from the center of the cell $\left(x_{k}=0,y_{k}=0\right)$ with a zero speed $\left(v_{x_{k}}=0,v_{y_{k}}=0\right)$, the goal is to reach at a fixed control time $t_{f}$ a given radius $r_{f}$ and phase $\varphi_{f}$. As an illustrative example, we force the phase to vary linearly with ω, contrary to the standard result obtained with chirp pulses, where a quadratic phase dependence is observed (see Appendix B for details). We denote by $r_{k}\left(t\right)$ and $\varphi_{k}\left(t\right)$, respectively, the radius and the phase of ion *k* at time *t*. We assume in a first step that there is no constraint on the electric field. A limitation on the maximum pulse intensity is accounted for in Section 3.2.

To simplify the notations, we omit below the index *k*. Using Equation (2), it is straightforward to show that $\Omega =\omega x+v_{y}$ is a constant of motion. At t=0, since x(0)=0 and $v_{y}\left(0\right)=0$, we deduce that Ω=0 so $v_{y}\left(t\right)=-\omega x\left(t\right)$. One of the two coordinates $v_{y}\left(t\right)$ or x(t) can be eliminated. This also means that we cannot control simultaneously the position and the speed of the ion with only one control. We arrive at:

$$\left\{\begin{array}{c} \dot{y}=v_{y}\\ {\dot{v}}_{y}=-\omega^{2}V_{x}\\ {\dot{V}}_{x}=v_{y}+e_{x} \end{array}\right.$$

where $V_{x}=v_{x}/\omega$. We introduce the vector $X=(y,v_{y},V_{x})$ whose dynamics are governed by:

(3) $$\dot{X}=AX+Ce_{x},$$

with

$$A=\left(\begin{array}{ccc} 0 & 1 & 0\\ 0 & 0 & -\omega^{2}\\ 0 & 1 & 0 \end{array}\right),C=\left(\begin{array}{c} 0\\ 0\\ 1 \end{array}\right)$$

The dynamics of this linear system can be explicitly integrated as follows. The eigenvalues of *A* are (0,iω,−iω) and the corresponding eigenvectors can be written as:

$$X_{0}=\left(\begin{array}{c} 1\\ 0\\ 0 \end{array}\right),X_{+}=\left(\begin{array}{c} 1\\ i\omega \\ 1 \end{array}\right),X_{-}=\left(\begin{array}{c} 1\\ -i\omega \\ 1 \end{array}\right)$$

At time $t_{f}$, the state of the system is given by:

$$X\left(t_{f}\right)=\int_{0}^{t_{f}}e^{A(t_{f}-s)}Ce_{x}\left(s\right)ds.$$

We have:

$$e^{At}=Pe^{Dt}P^{-1},$$

where D=diag(0,iω,−iω) and

$$P=\left(\begin{array}{ccc} 1 & 1 & 1\\ 0 & i\omega & -i\omega \\ 0 & 1 & 1 \end{array}\right),P^{-1}=\left(\begin{array}{ccc} 1 & 0 & -1\\ 0 & -0.5i/\omega & 0.5\\ 0 & 0.5i/\omega & 0.5 \end{array}\right)$$

We deduce that:

$$e^{At}=\left(\begin{array}{ccc} 1 & \sin(\omega t)/\omega & -1+\cos(\omega t)\\ 0 & \cos(\omega t) & -\omega \sin(\omega t)\\ 0 & \sin(\omega t)/\omega & \cos(\omega t) \end{array}\right)$$

and

(4) $$X\left(t_{f}\right)=\int_{0}^{t_{f}}dse_{x}\left(s\right)\left(\begin{array}{c} -1+\cos[\omega \left(t_{f}-s\right)]\\ -\omega \sin[\omega \left(t_{f}-s\right)]\\ \cos[\omega \left(t_{f}-s\right)] \end{array}\right)$$

### 2.2. The Rotating Wave Approximation

The oscillating excitation field $e_{x}$ applied only along the *x*- axis can be expressed as the sum of two rotating fields, one in the same direction as the ions and the other in the opposite direction. We introduce the Rotating Wave Approximation (RWA) which assumes that the field rotating in opposite direction to the ions has a negligible effect on their trajectories. This approximation is verified if the range of frequencies around the central frequency $\omega_{o}$ is not too large, as discussed in Appendix A. Note that RWA is a standard tool in NMR [20,52,53] where it is derived in a similar but different way due to the non-linearity of the system [54]. In particular for ICR, this approximation does not depend on the amplitude of the excitation. Using RWA, we show below that the control of ions is equivalent to the control of an ensemble of springs of different frequencies [47,51].

The derivation starts with the control of speeds which fulfill:

$$\left\{\begin{array}{c} {\dot{v}}_{xk}=\omega_{k}v_{yk}+\omega_{k}e_{x}\\ {\dot{v}}_{yk}=-\omega_{k}v_{xk} \end{array}\right.$$

In complex coordinates, we have:

(5) $${\dot{v}}_{k}=-i\omega_{k}v_{k}+\omega_{k}e_{x}\left(t\right),$$

where $v_{k}=v_{xk}+iv_{yk}$. We consider that $\omega_{k}\in \left[\omega_{0}-\delta \omega ,\omega_{0}+\delta \omega \right]$ where $\omega_{0}$ is the carrier frequency of the electric field, $e_{x}\left(t\right)=e_{0}\left(t\right)\cos\left(\omega_{0}t+\phi \left(t\right)\right)$, and δω is small compared to $\omega_{0}$. We also assume that the amplitude $e_{0}\left(t\right)$ and the phase ϕ(t) vary slowly in time with respect to the frequency $\omega_{0}$. We express the speed as: $v_{k}={\tilde{v}}_{k}e^{-i\omega_{0}t}$, where ${\tilde{v}}_{k}$ is the complex speed in the frame rotating at frequency $\omega_{0}$. We deduce that:

$${\dot{\tilde{v}}}_{k}=-i\Delta \omega_{k}{\tilde{v}}_{k}+\omega_{k}\frac{e_{0}}{2}\left(e^{-i\phi }+e^{2i\omega_{0}t+i\phi }\right),$$

where $\Delta \omega_{k}=\omega_{k}-\omega_{0}$ is the detuning term. In the RWA, we neglect the rapidly oscillating term $\exp(2i\omega_{0}t)$ and we arrive at:

(6) $${\dot{\tilde{v}}}_{k}≃-i\Delta \omega_{k}{\tilde{v}}_{k}+\omega_{k}\frac{e_{0}}{2}e^{-i\phi }.$$

It is worth noting here that, in the rotating frame, the dynamics are driven by two control parameters, $e_{0}\cos\phi$ and $e_{0}\sin\phi$. Note that we recover the control of an ensemble of springs. An additional step can be done for the position of the ion *k*, $x_{k}=x_{k}+iy_{k}$. We set $x_{k}={\tilde{x}}_{k}e^{-i\omega_{0}t}$. It is then straightforward to show that:

$${\dot{\tilde{x}}}_{k}-i\omega_{0}{\tilde{x}}_{k}={\tilde{v}}_{k}\left(t\right)$$

Since ${\tilde{x}}_{k}$ varies slowly with respect to $e^{i\omega_{0}t}$, we can neglect the time derivative ${\dot{\tilde{x}}}_{k}$, which gives:

$${\tilde{x}}_{k}=\frac{i}{\omega_{0}}{\tilde{v}}_{k}\left(t\right).$$

If the RWA is valid, we deduce that the speed control leads also to the control of the position of ions. In this study, the validity of RWA is verified in the different examples by a numerical integration of Equation (4).

## 3. Optimal Control Theory

### 3.1. A Short Introduction to Optimal Control Theory

We briefly introduce in this section the tools of optimal control theory used in this paper. To keep the introduction as accessible as possible, some mathematical details are not specified. We refer the interested reader to the specialized literature on the subject [1,2,10,13]. We consider a control system described by the following differential equation:

$$\dot{q}\left(t\right)=f\left(q\left(t\right),u\left(t\right)\right),$$

where $q\left(t\right)\in R^{n}$ is the state of the system at time *t*, *f* a smooth function and u(t)∈R the control law. We assume here that there is no constraint on the control amplitude. The goal of a control problem is to bring the state of the system from the initial state $q\left(0\right)=q_{0}$ as close as possible to a target state $q_{f}$ in a time $t_{f}$ while minimizing a given cost functional J. For a distance to the target state defined by $||q\left(t_{f}\right)-q_{f}\left|\right|$, a standard functional is:

$$J=\frac{1}{2}||q\left(t_{f}\right)-q_{f}{\left|\right|}^{2}+\lambda \int_{0}^{t_{f}}u{\left(t\right)}^{2}dt,$$

where λ is a positive constant which expresses the relative weight between the distance to the target state and the second term. This latter can be interpreted as the energy of the control. We formulate the optimal control from the Pontryagin Maximum Principle (PMP) which gives necessary conditions for a control solution to be optimal [2,4,9,11]. We introduce the Pontryagin Hamiltonian $H_{P}$ (the index *P* corresponds to Pontryagin) as:

$$H_{P}=p\left(t\right)\cdot f\left(q\left(t\right),u\left(t\right)\right)-\frac{\lambda u{\left(t\right)}^{2}}{2},$$

where $p\left(t\right)\in R^{n}$ is the adjoint state. This state plays qualitatively the role of a Lagrange multiplier for the optimization problem [10,13]. The state and the adjoint state of the dynamics fulfill the Hamilton’s equation:

$$\left\{\begin{array}{c} \dot{q}=\frac{\partial H_{P}}{\partial p}=f\left(q,u\right)\\ \dot{p}=-\frac{\partial H_{P}}{\partial q}=-p\cdot \frac{\partial f(q,u)}{\partial q} \end{array}\right.$$

with the following initial and final conditions q(0)=0 and $p\left(t_{f}\right)=-\frac{\partial J}{\partial q(t_{f})}=q_{f}-q\left(t_{f}\right)$, while the optimal control $u^{*}$ is given by $\frac{\partial H_{p}}{\partial u}=0$, i.e.,

$$u^{*}=\frac{p}{\lambda }\cdot \frac{\partial f(q,u^{*})}{\partial u}.$$

In the non-linear case, these conditions can be solved only for simple low-dimensional systems [4,11,25] and numerical algorithms are used for more complex dynamics [1,30,55]. For linear systems, the optimal solutions can be derived explicitly if there is no additional constraint on the control field. This approach is known in the literature as Linear Quadratic Optimal Control [2,10,47] and is applied to ICR in Appendix D. When experimental limitations such as maximum pulse intensity are accounted for in the numerical optimization process, the optimal control law is derived numerically from iterative algorithms, which are described in Section 3.2.

### 3.2. Optimal Gradient-Based Algorithm

The goal of this section is to develop a first-order gradient-based algorithm suited to this control problem [1]. We use a numerical optimization algorithm to take into account field amplitude constraint of the form $|e_{x}\left(t\right)|\leq e_{max}$. Note that this algorithm can be seen as the counterpart of the GRAPE algorithm in NMR [30] and that other limitations such as spectral constraints or bandwidth limitations could be added [56,57,58,59,60,61,62,63]. In the numerical simulations, the control field is described as a piece-wise constant function. Rapid time variations leading to high frequencies may appear in the optimization process. For question of numerical stability and precision, we apply the algorithm in the system with the RWA and then we use the derived control law in the original dynamical system.

We start from the differential system (6) written in the rotating frame for the ion *k* as:

$$\left\{\begin{array}{c} {\dot{\tilde{v}}}_{x}^{\left(k\right)}=\Delta \omega_{k}{\tilde{v}}_{y}^{\left(k\right)}+u_{x}\\ {\dot{\tilde{v}}}_{y}^{\left(k\right)}=-\Delta \omega_{k}{\tilde{v}}_{x}^{\left(k\right)}+u_{y} \end{array}\right.$$

where $u_{x}=\frac{\omega_{0}}{2}e_{0}\cos\phi$ and $u_{y}=-\frac{\omega_{0}}{2}e_{0}\sin\phi$. The two controls satisfy the limitation $u_{x}{\left(t\right)}^{2}+u_{y}{\left(t\right)}^{2}\leq u_{max}^{2}$ with $u_{max}=\frac{\omega_{0}}{2}e_{max}$. The corresponding target state is $\left({\tilde{v}}_{xf}^{\left(k\right)},{\tilde{v}}_{yf}^{\left(k\right)}\right)$. We consider a cost functional J with no penalty on the control field defined as:

(7) $$J=\frac{1}{2}\sum_{k}\left[{\left({\tilde{v}}_{xf}^{\left(k\right)}-{\tilde{v}}_{x}^{\left(k\right)}\left(t_{f}\right)\right)}^{2}+{\left({\tilde{v}}_{yf}^{\left(k\right)}-{\tilde{v}}_{y}^{\left(k\right)}\left(t_{f}\right)\right)}^{2}\right].$$

The Pontryagin Hamiltonian can be expressed as:

$$H_{P}=\sum_{k}\left[\Delta \omega_{k}\left(-p_{y}^{\left(k\right)}{\tilde{v}}_{x}^{\left(k\right)}+p_{x}^{\left(k\right)}{\tilde{v}}_{y}^{\left(k\right)}\right)+u_{x}p_{x}^{\left(k\right)}+u_{y}p_{y}^{\left(k\right)}\right].$$

The adjoint states fulfill the following relations:

(8) $$\left\{\begin{array}{c} {\dot{p}}_{x}^{\left(k\right)}=\Delta \omega_{k}p_{y}^{\left(k\right)}\\ {\dot{p}}_{y}^{\left(k\right)}=-\Delta \omega_{k}p_{x}^{\left(k\right)}. \end{array}\right.$$

The gradients are given by:

$$\frac{\partial H_{P}}{\partial u_{x}}=\sum_{k}p_{x}^{\left(k\right)},\frac{\partial H_{P}}{\partial u_{y}}=\sum_{k}p_{y}^{\left(k\right)}$$

The correction to the control fields $\delta u_{x}\left(t\right)$ and $\delta u_{y}\left(t\right)$ at each step of the algorithm is proportional to these gradients [1]. The final adjoint states can be expressed as:

$$\left\{\begin{array}{c} p_{x}^{\left(k\right)}\left(t_{f}\right)={\tilde{v}}_{xf}^{\left(k\right)}-{\tilde{v}}_{x}^{\left(k\right)}\left(t_{f}\right)\\ p_{y}^{\left(k\right)}\left(t_{f}\right)={\tilde{v}}_{yf}^{\left(k\right)}-{\tilde{v}}_{y}^{\left(k\right)}\left(t_{f}\right). \end{array}\right.$$

and Equation (8) can be directly integrated backward in time. We thus consider the following gradient-based algorithm.

1. Choose guess fields $u_{x}\left(t\right)$ and $u_{y}\left(t\right)$.
2. Propagate forward the state of every ion *k* and compute $\left(v_{x}^{\left(k\right)}\left(t_{f}\right),v_{y}^{\left(k\right)}\left(t_{f}\right)\right)$.
3. Propagate backward the adjoint state of the system from Equation (8).
4. Compute the corrections $\delta u_{x}\left(t\right)$ and $\delta u_{y}\left(t\right)$ to the control fields, $\delta u_{x}\left(t\right)=\epsilon \sum_{k}p_{x}^{\left(k\right)}$, $\delta u_{y}\left(t\right)=\epsilon \sum_{k}p_{y}^{\left(k\right)}$ where ϵ is a small positive constant.
5. Define the new control fields $u_{x}\left(t\right)\mapsto u_{x}\left(t\right)+\delta u_{x}\left(t\right)$$u_{y}\left(t\right)\mapsto u_{y}\left(t\right)+\delta u_{y}\left(t\right)$.
6. Truncate the new control fields $u_{x}\left(t\right)$ and $u_{y}\left(t\right)$ to satisfy the constraint $\sqrt{u_{x}{\left(t\right)}^{2}+u_{y}{\left(t\right)}^{2}}\leq u_{max}$:
  $$u_{x}\left(t\right)\mapsto \frac{u_{x}\left(t\right)u_{max}}{\sqrt{u_{x}{\left(t\right)}^{2}+u_{y}{\left(t\right)}^{2}}},u_{y}\left(t\right)\mapsto \frac{u_{y}\left(t\right)u_{max}}{\sqrt{u_{x}{\left(t\right)}^{2}+u_{y}{\left(t\right)}^{2}}}.$$
7. Go to Step 2 until a given accuracy is reached.

Similar algorithms are used in NMR for taking into account pulse constraints [15,16,17]. Note that the use of a gradient causes this type of algorithm to converge towards a local maximum of the optimization problem. Numerical simulations with different guess fields allow partly overcoming this limitation, even if the global maximum is not reached with certainty. The efficiency of this algorithm in ICR is illustrated numerically in Section 4.

## 4. Numerical Results

We present numerical results obtained either with LQOCT (see Appendix D for details) or with the gradient-based algorithm. A comparison with the SWIFT approach described in Appendix C is also done. Different experimental constraints have to be satisfied by the control pulse. The objective is to excite ions in a wide range of frequencies around a central frequency of the order of 500 kHz. The excitation has to be as uniform as possible in radius and phase in the range $\left[f_{min},f_{max}\right]$ and close to zero outside. As a benchmark example, we choose in this section to consider the interval [400, 600] kHz. Using the linearity of the dynamics, these results can be transposed to another range of frequencies by a scaling of the excitation pulse duration and of the pulse amplitude. For instance, if the total process time is increased by a factor α, then the range of frequencies and the amplitude of the electric field are divided by the same parameter α. The description of the optimal control of this infinite dimensional dynamical system is mathematically quite intricate, even if some results can be established [47,50]. For practical and numerical reasons, it is more convenient to consider a finite set of *N* systems by discretizing the frequency interval. We thus consider the simultaneous control of each element of this set. In this paper, we consider a regular discretization, but other choices could be possible, and the frequency step is chosen small enough to avoid the discretization effect. Note that the same approach is used in NMR to control a spin ensemble [15,16,17]. The required sharp excitation profile is modeled by the following function:

$$r\left(f\right)=\frac{r_{0}}{2}\left[\tanh\left(\mu \left(f-f_{min}\right)\right)+\tanh\left(\mu \left(f_{max}-f\right)\right)\right],$$

where μ is a free parameter allowing to adjust the slope of the excitation gate. In the numerical simulations, μ is fixed to 0.1. We impose that the final radius is $r_{f}=5$ cm and a final phase varying linearly with the frequency, with a maximum variation of the order of 1${}^{\circ }$/1 Hz, which corresponds to 17.45 rad/kHz. The final phase $\varphi_{f}^{\left(k\right)}$ of the ion *k* is expressed as $\varphi_{f}^{\left(k\right)}=-a\omega_{k}t_{f}$, where *a* is a parameter characterizing the slope of the angular variation. The magnetic field is set to 7 T and the maximum electric field amplitude that can be generated is of the order of $10^{3}$ V·m${}^{-1}$. The control time can be very long, of the order of few hundred ms, but more stable numerical results were achieved for duration of the order of few ms.

**Figure 1:**
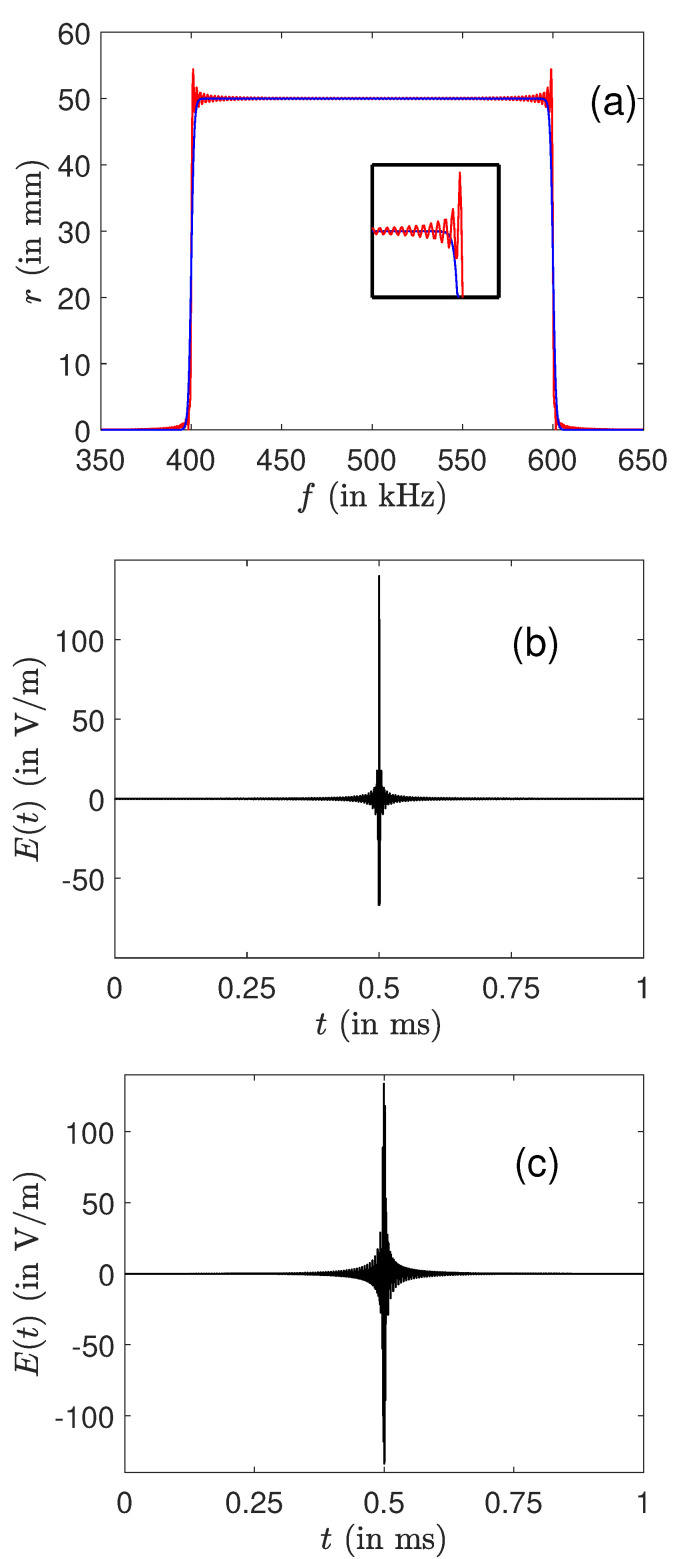

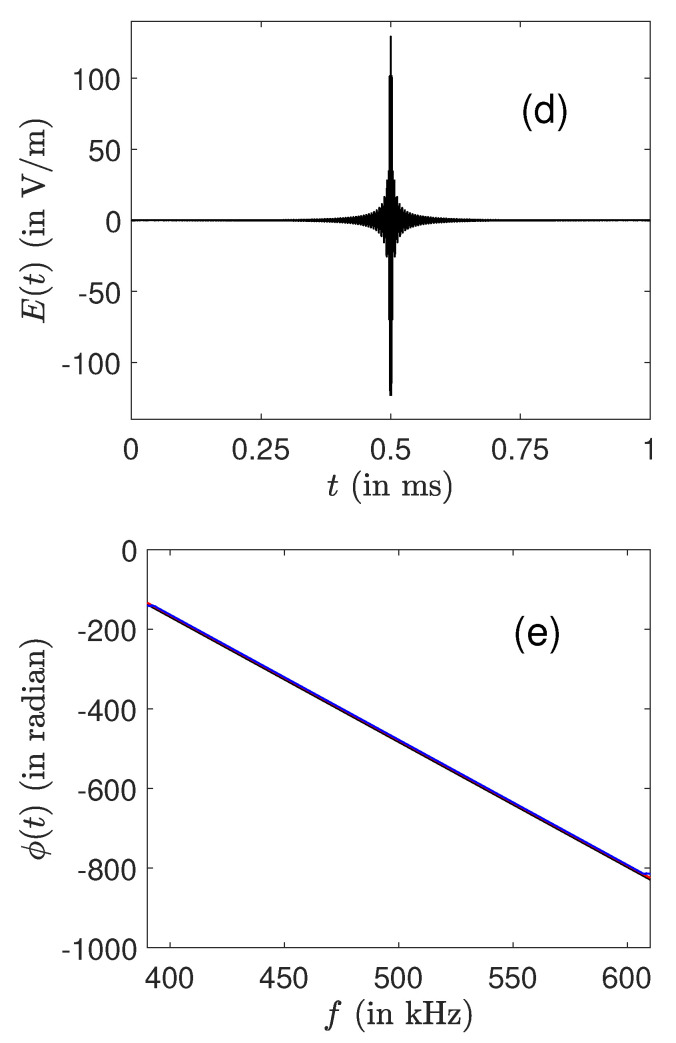
Comparison between the optimal and the SWIFT approaches for the robust control of an ensemble of ions in the frequency range [400,600] kHz. The small insert is a zoom of the profile around the frequency f=600 kHz. (**a**,**e**) The evolution of the final radius and phase as a function of *f*. Note that an arbitrary constant has been added to the phase in order to superimpose the curves (the three lines are practically indistinguishable in (**e**)). The black, blue (dark gray) and red (light gray) solid lines depict, respectively, the optimal solutions computed without and with the RWA and the SWIFT pulse. The SWIFT and optimal control laws are plotted in (**b**,**c**) (optimal without RWA) and (**d**) (optimal with RWA). The number of discretized frequency points is set to 601 in the optimization process in the range [350,650] kHz.

We first present in Figure 1a series of simulations without limitation on the field intensity. The excitation pulse duration is chosen to be equal to 1 ms. The electric field is computed from a set of the order of 500 frequencies regularly spaced in the interval under study. Since the derived solutions are very sharp, this duration can be modified to some extent without changing the control pulse. Figure 1 compares the results achieved by LQOCT and by the SWIFT approach. The optimal solutions can be computed by using or not the RWA. Note that the pulse computed in the RWA is then applied without any approximation to the original system. In the case displayed in Figure 1, very similar efficiencies are obtained for the two optimal excitations. The optimal pulses have a shape similar to that of the SWIFT solution, even if their amplitudes and durations are different. We recall that the analytical expressions of the pulses are different, but that, for a continuous set of frequencies, the control field is expected to be unique. This statement can be rigorously shown in the case of an ensemble of springs under some specific mathematical assumptions [47,50]. However, the optimal control method offers greater flexibility since one can play with different parameters such as the cost functional or the number of discrete frequencies to adjust the final result.

**Figure 2:**
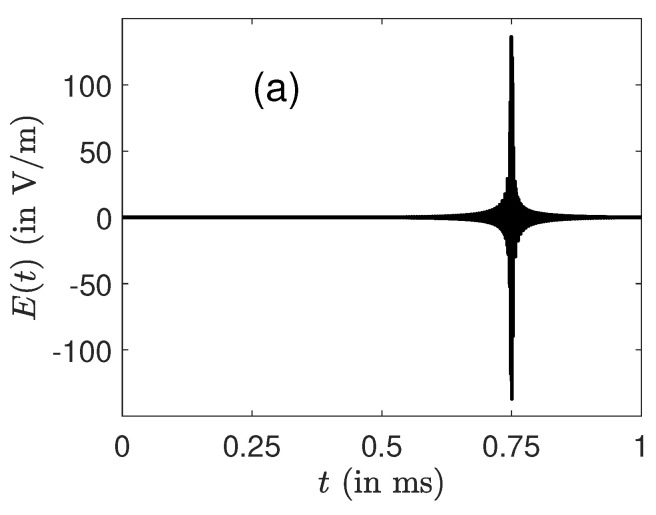

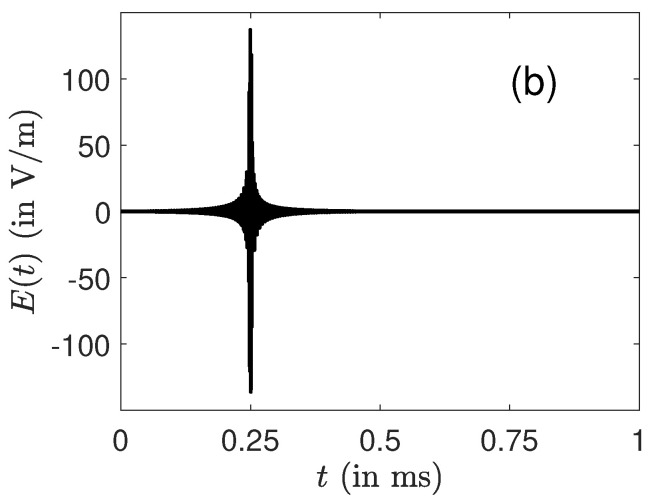
Same as Figure 1 but for different slopes of the excitation profile. The parameter *a* is fixed, respectively, to 0.25 and 0.75 in (**a**,**b**). The optimal pulses without RWA are represented.

We study in Figure 2 the role of the phase slope of the excitation profile on the structure of the pulse. Figure 2 shows that this slope changes the position of the peak of the pulse. This position can be deduced from a Fourier transform of the profile. Very good results were obtained for slopes in the range [0.05, 0.95] with a maximum pulse amplitude almost constant. Pulse distortion appears when the slope parameter *a* is close to 0 or 1. For a=0, it becomes very difficult to control all the ions which have to reach a fixed target state in a space-fixed frame, independently of their own frequency. Note that similar results were achieved in NMR [64,65], which highlights the similarities between the control of the two dynamics.

**Figure 3:**
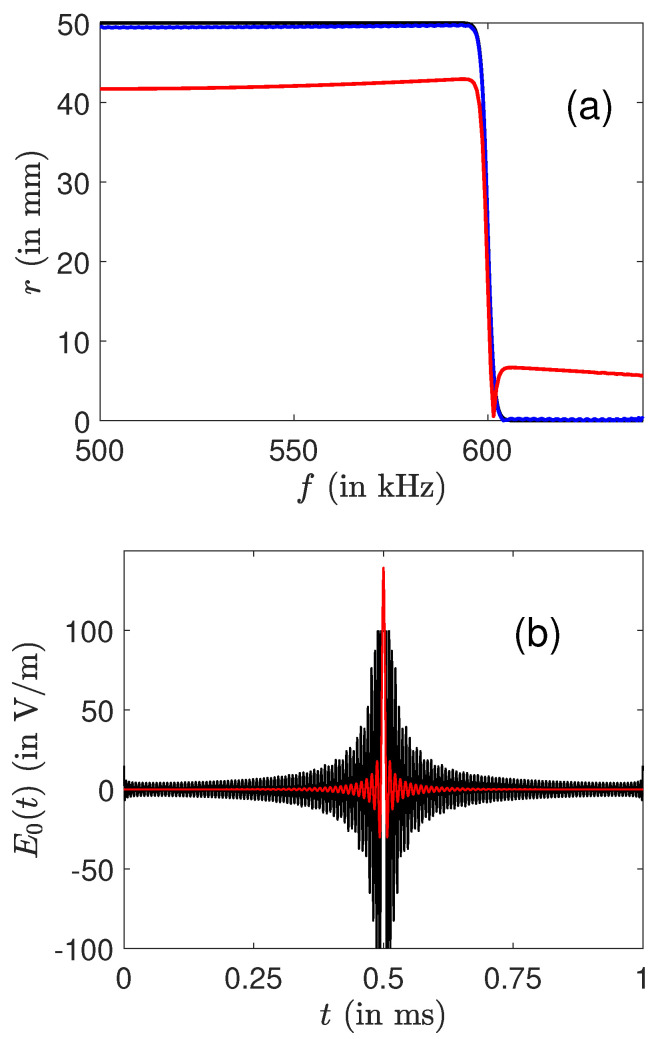
(**a**) The final radius *r* as a function of the frequency *f*. The black, blue (dark gray) and red (light gray) curves represent, respectively, the ideal profile, the one obtained with the optimization algorithm and the one corresponding to the optimal pulse of Figure 1 whose amplitude has been abruptly limited (see the text for details). Note that the black and blue lines in (**a**) are almost superimposed. The amplitude $E_{0}$ of the optimal fields in the rotating frame with (black curve) and without (red or light gray curve) constraints are depicted in (**b**).

We now focus on ion control with amplitude constraint. The numerical simulations were carried out by assuming the RWA. The same set of discretized frequencies is chosen. We optimize piecewise constant functions with a time step lower than 1 μs to avoid discretization effect. The dynamics are integrated numerically through the formulas given in Section 2. More than 1000 iterations are usually needed to converge to an efficient solution. In a first step, we apply the gradient-based algorithm described in Section 3.2 with only one control field, namely $E_{0}\left(t\right)=e_{0}\left(t\right)B$, and the phase ϕ(t) of the electric field is set to 0. We consider the same control problem as before and the optimal solutions derived above are used as guess field for the optimization algorithm. Figure 3 displays the best result achieved with this limitation. The maximum field amplitude can be reduced from 130 to 100 V·m${}^{-1}$ while maintaining an almost ideal excitation profile. This reduction was made possible by a distribution of the energy along the control interval. Outside of t≃0.5 ms, the amplitude of the optimized field is much larger than the one of the guess pulse. As a comparison, Figure 3 also presents the profile obtained from the optimal pulse of Figure 1 whose amplitude has been arbitrarily limited to 100 V·m${}^{-1}$, showing the non-trivial transformation made by the algorithm.

**Figure 4:**
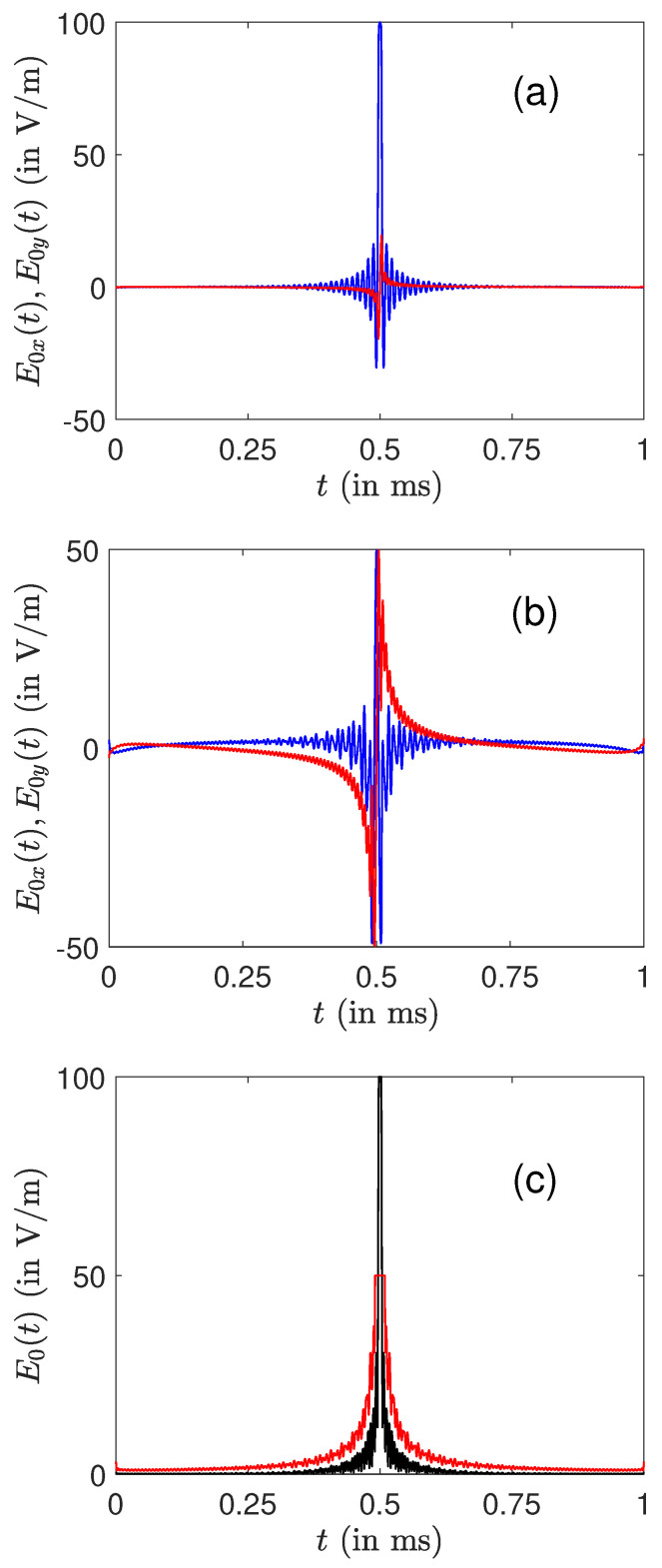
Plot of the optimal amplitudes $E_{0x}$ (blue or dark gray) and $E_{0y}$ (red or light gray) for a maximum amplitude of 100 V·m${}^{-1}$ (**a**)) and 50 V·m${}^{-1}$ (**b**). (**c**) The corresponding total amplitude $E_{0}=\sqrt{E_{0x}^{2}+E_{0y}^{2}}$.

The optimization algorithm fails to converge towards a very good excitation profile, when the maximum amplitude is much smaller than 100 V·m${}^{-1}$. Note that more advanced optimal algorithms could be tested in this case to verify these convergence issues [66]. This obstacle can be partly overcome by considering in a second step two control fields (in the rotating frame) denoted $E_{0x}=e_{0}B\cos\phi$ and $E_{0y}=e_{0}B\sin\phi$. An example is displayed in Figure 4 for a maximum amplitude of 100 and 50 V·m${}^{-1}$. An almost perfect excitation profile is achieved in these two cases. Note the different structures of the fields along the *x*- and *y*-directions, namely even and odd functions. This observation was also made in some optimal control problems in NMR [17].

**Figure 5:**
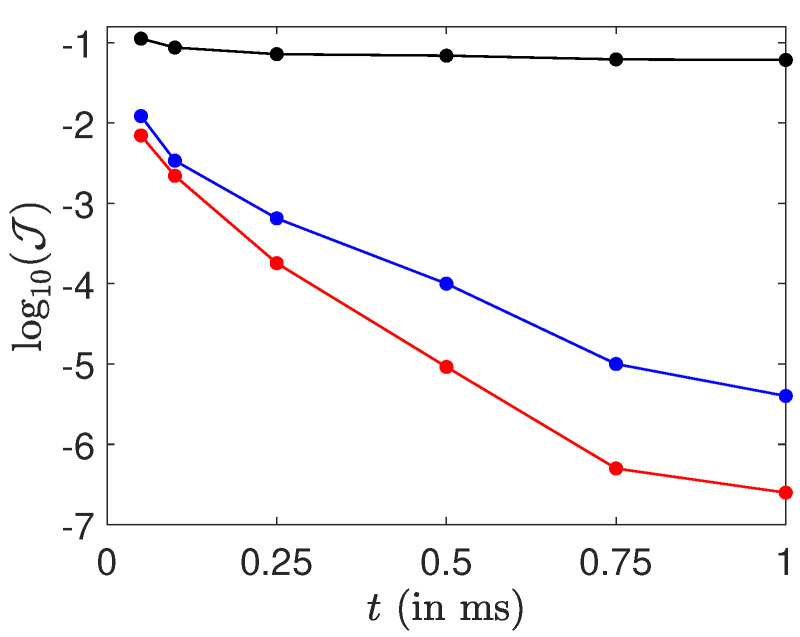
Evolution of the logarithm of the cost functional J as a function of the control time for different maximum amplitudes (black, 30 V·m${}^{-1}$; blue or dark gray, 50 V·m${}^{-1}$; red or light gray, 70 V·m${}^{-1}$).

A systematic analysis of the efficiency of the optimized control fields with respect to the maximum pulse amplitude and to the control duration is provided in Figure 5. The efficiency of the control process is measured from the cost functional J given in Equation (7). As could be expected, we observe that better results are achieved for larger maximum amplitude and control time. However, the final fidelity varies in a quite complex way with the control time. A saturation is observed for times of the order of few milliseconds. It is not clear if this point is due to an intrinsic limitation of the control protocol or to convergence problems of the algorithm. Further investigations are needed to clarify this issue.

## 5. Conclusions

We applied optimal control techniques to the robust excitation of ions in ICR. We considered the simplified but realistic conditions of a two-dimensional trajectory and of a homogeneous magnetic field. In this model system, we propose different ways to solve the optimal control problems. Such methods are directly inspired from NMR in which OCT is a standard and efficient tool. In the case without pulse limitation, the linearity of the dynamical equations allows using LQOCT, which has the advantage to lead to an analytical formula of the control law. Very good results were obtained both for the final radii and phases of the ions. A specific range of frequencies was considered in this study, but the same approach can be extended to broadband excitation from 100 to 900 kHz. However, this solution is both in shape and in amplitude very similar to the SWIFT pulse. The two solutions are expected to be equal for a continuous range of frequencies. More original control laws are derived when the pulse intensity is limited. Due to this constraint, optimal iterative algorithms have to be used, and we adapt to ICR the standard GRAPE algorithm, well-known in NMR. Even if this algorithm has some limitations, it allows reducing the pulse intensity, by a factor larger than three in the examples under study. On the basis of NMR results, this algorithm is expected to be very efficient in the case of other excitation profiles. The very encouraging and promising results obtained in this investigation must now be confirmed by experimental implementation. Numerical simulations of this study are not fully realistic. Effects such as the magnetron motion, field geometry, field inhomogeneities or ion collisions are neglected. However, the model system we consider describes quite faithfully the main cyclotronic behavior and permits to grasp rapidly the main features of ion trajectories. Numerical codes were developed to account for such experimental details. The relative simplicity of the application of numerical optimal algorithms makes it possible to adapt it straightforwardly to a new class of control problems. They could thus be combined with such codes. We are therefore quite confident about the extension of optimization procedures to these additional experimental constraints and limitations. Work is in progress on these different issues.

All pulse shapes are available upon request to the corresponding author.

## Figures and Tables

**Figure A1:**
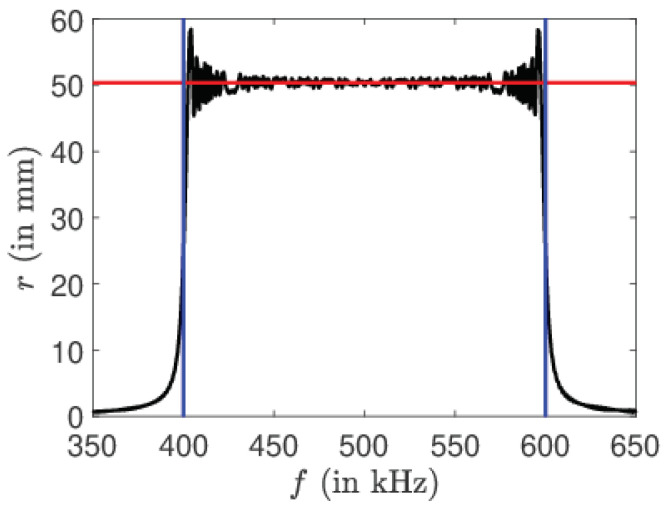
Excitation of an ensemble of ions by an adiabatic pulse: Evolution of the final radius as a function of the frequency. The parameters are set to $t_{f}=10$ ms, $E_{0}$ = 3.2 V/m and $B_{0}$ = 7 T. The red (light gray) solid line represents the stationary phase approximation. The vertical blue (dark gray) solid lines indicate the range of frequency of the pulse.

## Data Availability

The data presented in this study are available in article.

## Abbreviations

The following abbreviations are used in this manuscript:

OCT	Optimal Control Theory
LQOCT	Linear Quadratic Optimal Control Theory
ICR	Ion Cyclotron Resonance
PMP	Pontryagin Maximum Principle
NMR	Nuclear Magnetic Resonance
RWA	Rotating Wave Approximation

## Appendix A. The Rotating Wave Approximation

We discuss in this section the validity of the Rotating Wave Approximation described in Section 2.2. We consider the following dynamical system:

(A1) $$\dot{z}=-i\omega z+u\cos\left(\omega_{0}t\right)$$

which corresponds to Equation (5) of the main text. Equation (A1) describes a spring of frequency ω/(2π) excited by an external field of constant amplitude *u* and of frequency $\omega_{0}/\left(2\pi \right)$. Introducing the frame rotating at $\omega_{0}$ with the transformation $z=\tilde{z}e^{-i\omega_{0}t}$, we arrive at:

$$\dot{\tilde{z}}=-i\Delta \omega \tilde{z}+\frac{u}{2}\left(1+e^{2i\omega_{0}t}\right),$$

where $\Delta \omega =\omega -\omega_{0}$ is the detuning. In the RWA, we neglect the fast oscillating term and we get:

$${\dot{\tilde{z}}}_{r}=-i\Delta \omega {\tilde{z}}_{r}+\frac{u}{2}.$$

where ${\tilde{z}}_{r}$ denotes the approximate $\tilde{z}$- variable. We set $\delta \tilde{z}=\tilde{z}-{\tilde{z}}_{r}$ and obtain:

$$\delta \dot{\tilde{z}}=-i\Delta \omega \delta \tilde{z}+\frac{u}{2}e^{2i\omega_{0}t}.$$

This differential system can be exactly integrated:

$$\delta \tilde{z}\left(t\right)=\int_{0}^{t}e^{-i\Delta \omega (t-\tau )}\frac{u}{2}e^{2i\omega_{0}\tau }d\tau .$$

This leads to:

$$\delta \tilde{z}\left(t\right)=e^{i(\omega_{0}-\frac{\Delta \omega }{2})t}\frac{u}{2\omega_{0}+\Delta \omega }\sin\left(\left(\omega_{0}+\frac{\Delta \omega }{2}\right)t\right).$$

We deduce that the relative error due to the RWA can be expressed as:

$$|\frac{\delta \tilde{z}}{{\tilde{z}}_{r}}|=\frac{\left|\Delta \omega \right|}{2\omega_{0}+\Delta \omega }\left|\frac{\sin(\left(\omega_{0}+\Delta \omega /2\right)t)}{\sin(\Delta \omega t/2)}\right|.$$

A rough approximation gives:

$$|\frac{\delta \tilde{z}}{{\tilde{z}}_{r}}|≃\frac{\left|\Delta \omega \right|}{2\omega_{0}+\Delta \omega }$$

RWA is therefore justified if $\left|\Delta \omega \right|\ll 2\omega_{0}$. Numerical simulations show that this formula overestimates the error and that RWA can be used in a quite wide interval around the carrier frequency of the excitation pulse.

## Appendix B. Adiabatic Excitation of ICR Process

The goal of this paragraph is to compute the final states of the ions in the case of an adiabatic excitation of the form $e_{x}=e_{0}\cos\left(\omega_{i}t+\frac{s}{2}t^{2}\right)$ where $\omega_{i}$ is the initial frequency and *s* the sweep rate. We recall that integrals of the form

$$I\left(\alpha ,\beta \right)=\int_{0}^{t_{f}}\exp\left[i\alpha t^{2}+i\beta t\right]dt$$

can be computed from the Erfi function. This result allows computing exactly the dynamics of the system. Starting from Equation (4), the final state of the ICR process can be expressed as follows:

$$X\left(t_{f}\right)=e_{0}\int_{0}^{t_{f}}dt\left(\begin{array}{c} -\cos\left(\omega_{i}t+st^{2}/2\right)+\cos\left(st^{2}/2+\left(\omega_{i}-\omega \right)t+\omega t_{f}\right)/2+\cos\left(st^{2}/2+\left(\omega_{i}+\omega \right)t-\omega t_{f}\right)/2\\ -\omega [\sin\left(st^{2}/2+\left(\omega_{i}-\omega \right)t+\omega t_{f}\right)/2-\sin\left(st^{2}/2+\left(\omega_{i}+\omega \right)t-\omega t_{f}\right)/2]\\ \cos\left(st^{2}/2+\left(\omega_{i}-\omega \right)t+\omega t_{f}\right)/2+\cos\left(st^{2}/2+\left(\omega_{i}+\omega \right)t-\omega t_{f}\right)/2 \end{array}\right)$$

and we finally obtain:

$$\left\{\begin{array}{c} x_{\omega }\left(t_{f}\right)/e_{0}=ℑ\left[\frac{e^{i\omega t_{f}}}{2}I\left(\frac{s}{2},\omega_{i}-\omega \right)-\frac{e^{-i\omega t_{f}}}{2}I\left(\frac{s}{2},\omega_{i}+\omega \right)\right]\\ y_{\omega }\left(t_{f}\right)/e_{0}=ℜ\left[-I\left(\frac{s}{2},\omega_{i}\right)+\frac{e^{i\omega t_{f}}}{2}I\left(\frac{s}{2},\omega_{i}-\omega \right)+\frac{e^{-i\omega t_{f}}}{2}I\left(\frac{s}{2},\omega_{i}+\omega \right)\right] \end{array}\right.$$

An approximation of the dynamics can be derived by using the stationary phase approximation. For that purpose, we start from Equation (5) and we assume that $\int_{0}^{t_{f}}e_{x}\left(t\right)dt=0$. We have:

$$v_{k}\left(t_{f}\right)=\omega_{k}e^{-i\omega_{k}t_{f}}\int_{0}^{t_{f}}dte_{x}\left(t\right)e^{i\omega_{k}t}.$$

The stationary phase approximation can be stated as follows. We consider the following integral:

$$\hat{h}\left(\omega \right)=\int_{-\infty }^{+\infty }h\left(t\right)e^{i\phi (t)}dt,$$

where ϕ is a smooth function, which is assumed to be rapidly varying with respect to *h*. A stationary point $t_{0}$ is defined by $\phi^{\left(1\right)}\left(t_{0}\right)=0$, where $\phi^{\left(n\right)}$ denotes the *n*th time derivative of ϕ. A Taylor expansion around $t=t_{0}$ leads to:

$$\phi \left(t\right)=\phi \left(t_{0}\right)+\left(t-t_{0}\right)\phi^{\left(1\right)}\left(t_{0}\right)+\frac{{\left(t-t_{0}\right)}^{2}}{2}\phi^{\left(2\right)}\left(t_{0}\right)+\cdots$$

We arrive at:

$$\begin{array}{ccc} \hat{h}\left(\omega \right) & ≃ & h\left(t_{0}\right)e^{i\phi (t_{0})}\int_{-\infty }^{+\infty }e^{i\frac{\xi^{2}}{2}\phi^{\left(2\right)}\left(t_{0}\right)}d\xi \\ & ≃ & \sqrt{\frac{2\pi }{\phi^{\left(2\right)}\left(t_{0}\right)}}h\left(t_{0}\right)e^{i(\phi \left(t_{0}\right)+\frac{\pi }{4})}. \end{array}$$

For a chirp excitation, the phase ϕ(t) is defined by $\phi \left(t\right)=\omega_{i}t+\frac{st^{2}}{2}$. The instantaneous frequency ω(t) can be expressed as:

$$\omega \left(t\right)=\dot{\phi }\left(t\right)=\omega_{i}+st,$$

where $s=\dot{\omega }\left(t\right)$. In the example under study, the rate *s* is given by $s=\left(\omega_{f}-\omega_{i}\right)/t_{f}$. We assume that s>0 and we deduce that the Fourier transform of the control field is given by:

$$\begin{array}{ccc} {\hat{e}}_{x}\left(\omega \right) & = & \int_{0}^{t_{f}}e_{x}\left(t\right)e^{i\omega t}dt\\ & = & \frac{e_{0}}{2}\int_{0}^{t_{f}}\left[e^{-i(\omega_{i}t+\frac{st^{2}}{2}-\omega t)}+e^{i(\omega_{i}t+\frac{st^{2}}{2}+\omega t)}\right]dt. \end{array}$$

We denote by $\phi_{1}$ and $\phi_{2}$ the arguments of the two exponential terms. It is straightforward to verify that ${\dot{\phi }}_{1}\left(t\right)=0$ for $t=t_{1}^{\left(\omega \right)}=\frac{\omega -\omega_{i}}{s}$ and that ${\dot{\phi }}_{2}\left(t\right)=0$ for $t=t_{2}^{\left(\omega \right)}=\frac{-\omega -\omega_{i}}{s}$. Neglecting the second contribution since $t_{2}^{\left(\omega \right)}<0$ and assuming that $t_{1}^{\left(\omega \right)}$ is not too close to 0 and $t_{f}$, we can consider that the integral is defined from −∞ to +∞. We finally get:

$${\hat{e}}_{x}\left(\omega \right)=e_{0}\sqrt{\frac{\pi }{2s}}e^{i(\frac{\pi }{4}+\phi_{1}\left(t_{1}^{\left(\omega \right)}\right))}.$$

The phase spectrum $\phi \left(\omega \right)=\frac{\pi }{4}+\phi_{1}\left(t_{1}^{\left(\omega \right)}\right)$ can be written as:

$$\phi \left(\omega \right)=\frac{\pi }{4}+\frac{{\left(\omega -\omega_{i}\right)}^{2}}{2s}.$$

Coming back to the original control problem, we obtain:

$$v_{k}\left(t_{f}\right)≃\omega_{k}e_{0}\sqrt{\frac{\pi }{2s}}\exp\left[i\left(\frac{\pi }{4}-\omega_{k}t_{f}+\frac{{\left(\omega_{k}-\omega_{i}\right)}^{2}}{2s}\right)\right].$$

In the range of validity of this approximation, we observe that the final radius of ions at time $t=t_{f}$ is a constant, while the phase varies quadratically with the frequency ω. A numerical example is given in Figure A1. The frequency of the chirped pulse goes from 400 to 600 kHz.

## Appendix C. Excitation by the SWIFT Approach

In this paragraph, we describe the application of the SWIFT method to the model system. We consider a specific approach in which the control law and the corresponding dynamics can be expressed analytically.

The dynamics are governed by the differential system (2). In the RWA described in Section 2.2, the dynamics can be approximated as:

$${\dot{\tilde{v}}}_{k}=-i\Delta \omega_{k}{\tilde{v}}_{k}+\frac{\omega_{0}}{2}e_{0}e^{-i\phi },$$

where ${\tilde{v}}_{k}={\tilde{v}}_{xk}+i{\tilde{v}}_{yk}$ and the control field is expressed as $e_{x}\left(t\right)=e_{0}\left(t\right)\cos\left(\omega_{0}t+\phi \left(t\right)\right)$. The differential equation can be integrated and leads to:

$${\tilde{v}}_{k}\left(t_{f}\right)=\int_{0}^{t_{f}}e^{-i\Delta \omega_{k}\left(t_{f}-t\right)}\frac{\omega_{0}}{2}e_{0}e^{-i\phi }dt$$

We deduce that:

$${\tilde{v}}_{k}^{*}\left(t_{f}\right)e^{-i\Delta \omega_{k}t_{f}}=\int_{0}^{t_{f}}e^{-i\Delta \omega_{k}t}\frac{\omega_{0}}{2}e_{0}e^{i\phi }dt.$$

Introducing $u\left(t\right)=e_{0}e^{i\phi }$ and assuming that *u* is different from zero only in the interval $\left[0,t_{f}\right]$, we obtain:

$$\sqrt{2\pi }\frac{\omega_{0}}{2}\hat{u}\left(\Delta \omega_{k}\right)={\tilde{v}}_{k}^{*}\left(t_{f}\right)e^{-i\Delta \omega_{k}t_{f}}.$$

where we use the following definition for the Fourier transform:

$$f\left(t\right)=\frac{1}{\sqrt{2\pi }}\int_{-\infty }^{+\infty }\hat{f}\left(\omega \right)e^{i\omega t}d\omega ;\hat{f}\left(\omega \right)=\frac{1}{\sqrt{2\pi }}\int_{-\infty }^{+\infty }f\left(t\right)e^{-i\omega t}dt.$$

The target states are defined as:

$$\left\{\begin{array}{c} r_{\Delta \omega_{k}}=r_{0}\Pi \left(\frac{\Delta \omega_{k}}{\delta \omega }\right)\\ \phi_{\Delta \omega_{k}}=a\Delta \omega_{k}+\phi_{0} \end{array}\right.$$

where Π is the gate function, with Π(x)=1 if $\left|x\right|\leq \frac{1}{2}$ and 0 otherwise. The parameter δω is the width of the distribution and $\phi_{0}$ is an arbitrary constant. We have:

$$x_{k}=r_{\Delta \omega_{k}}e^{i(a\Delta \omega_{k}+\phi_{0})}.$$

In the RWA, starting from ${\tilde{v}}_{k}=-i\omega_{0}{\tilde{x}}_{k}$, we arrive at:

$${\tilde{v}}_{k}\left(t\right)=-i\omega_{0}r_{\Delta \omega_{k}}e^{i(a\Delta \omega_{k}+\phi_{0})}e^{i\omega_{0}t}$$

and

$$\hat{u}\left(\Delta \omega \right)=\frac{2i}{\sqrt{2\pi }}r_{\Delta \omega_{k}}e^{-i\Delta \omega_{k}\left(t_{f}+a\right)}e^{-i\phi_{0}-i\omega_{0}t_{f}},$$

which gives

$$u\left(t\right){=\mathrm{FT}}^{-1}\left[\frac{2i}{\sqrt{2\pi }}r_{\Delta \omega_{k}}e^{-i\Delta \omega_{k}\left(t_{f}+a\right)}e^{-i\phi_{0}-i\omega_{0}t_{f}}\right].$$

Since

$$\frac{1}{\sqrt{2\pi }}\int_{-\infty }^{+\infty }\Pi \left(\frac{\omega }{\delta \omega }\right)e^{i\omega t}d\omega =\frac{\delta \omega }{\sqrt{2\pi }}\mathrm{sinc}\left(\frac{\delta \omega t}{2}\right),$$

we obtain:

$$u\left(t\right)=\frac{r_{0}\delta \omega e^{i\phi_{1}}}{\pi }\mathrm{sinc}\left[\frac{\delta \omega }{2}\left(t-t_{0}\right)\right],$$

with $t_{0}=t_{f}+a$ and $\phi_{1}$ an arbitrary phase. The original control field $e_{x}\left(t\right)=e_{0}\left(t\right)\cos\left(\omega_{0}t+\phi \left(t\right)\right)$ is then given by:

$$e_{x}\left(t\right)=\frac{r_{0}\delta \omega }{\pi }\mathrm{sinc}\left(\frac{\delta \omega }{2}\left(t-t_{0}\right)\right)\cos\left(\omega_{0}t+\phi_{1}\right).$$

Since the choice of the initial phase $\phi_{1}$ is arbitrary, we finally get:

$$e_{x}\left(t\right)=\frac{r_{0}\delta \omega }{\pi }\mathrm{sinc}\left(\frac{\delta \omega }{2}\left(t-t_{0}\right)\right)\cos\left(\omega_{0}\left(t_{f}-t\right)\right).$$

The next step consists in integrating exactly the system dynamics using the original system and Equation (4). We have to compute terms of the form:

$$\left\{\begin{array}{c} I_{c}\left(t,\omega_{s},\omega \right)=\int \mathrm{sinc}\left(\omega_{s}\left(t-t_{0}\right)\right)\cos\left(\omega \left(t_{f}-t\right)\right)dt\\ I_{s}\left(t,\omega_{s},\omega \right)=\int \mathrm{sinc}\left(\omega_{s}\left(t-t_{0}\right)\right)\sin\left(\omega \left(t_{f}-t\right)\right)dt. \end{array}\right.$$

For that purpose, we use the sine and the cosine integral functions Si and Ci, which are defined by:

$$\mathrm{Si}\left(x\right)=\int_{0}^{x}\mathrm{sinc}\left(t\right)dt,\mathrm{Ci}\left(x\right)=-\int_{x}^{\infty }\frac{\cos t}{t}dt,x>0.$$

We have the following results:

$$\begin{array}{ccc} I_{c}\left(t,\omega_{s},\omega \right) & = & \frac{\sin[\left(t_{0}-t_{f}\right)\omega ]}{2\omega_{s}}\left(\mathrm{Ci}\left[\left(t_{0}-t\right)\left(\omega +\omega_{s}\right)\right]-\mathrm{Ci}\left[\left(t_{0}-t\right)\left(\omega -\omega_{s}\right)\right]\right)+\\ & & \frac{\cos[\left(t_{0}-t_{f}\right)\omega ]}{2\omega_{s}}\left(\mathrm{Si}\left[\left(t_{0}-t\right)\left(\omega -\omega_{s}\right)\right]-\mathrm{Si}\left[\left(t_{0}-t\right)\left(\omega +\omega_{s}\right)\right]\right) \end{array}$$

$$\begin{array}{ccc} I_{s}\left(t,\omega_{s},\omega \right) & = & \frac{\cos[\left(t_{0}-t_{f}\right)\omega ]}{2\omega_{s}}\left(\mathrm{Ci}\left[\left(t_{0}-t\right)\left(\omega +\omega_{s}\right)\right]-\mathrm{Ci}\left[\left(t_{0}-t\right)\left(\omega -\omega_{s}\right)\right]\right)+\\ & & \frac{\sin[\left(t_{0}-t_{f}\right)\omega ]}{2\omega_{s}}\left(\mathrm{Si}\left[\left(t_{0}-t\right)\left(\omega +\omega_{s}\right)\right]-\mathrm{Si}\left[\left(t_{0}-t\right)\left(\omega -\omega_{s}\right)\right]\right) \end{array}$$

The final state of the dynamics is given by the following expressions:

$$\left\{\begin{array}{c} x_{\omega }\left(t_{f}\right)=\int_{0}^{t_{f}}dte_{x}\left(t\right)\sin\left[\omega \left(t_{f}-t\right)\right]\\ y_{\omega }\left(t_{f}\right)=\int_{0}^{t_{f}}dte_{x}\left(t\right)\left(-1+\cos\left[\omega \left(t_{f}-t\right)\right]\right). \end{array}\right.$$

We then deduce:

$$x_{\omega }\left(t_{f}\right)=\frac{r_{0}\delta \omega }{2\pi }\left[I_{s}\left(t_{f},\omega_{s},\omega_{0}+\omega \right)-I_{s}\left(0,\omega_{s},\omega_{0}+\omega \right)-I_{s}\left(t_{f},\omega_{s},\omega_{0}-\omega \right)+I_{s}\left(0,\omega_{s},\omega_{0}-\omega \right)\right]$$

and

$$\begin{array}{ccc} y_{\omega }\left(t_{f}\right) & = & \frac{r_{0}\delta \omega }{2\pi }[-2I_{c}\left(t_{f},\omega_{s},\omega_{0}\right)+2I_{c}\left(0,\omega_{s},\omega_{0}\right)\\ & & +I_{c}\left(t_{f},\omega_{s},\omega_{0}+\omega \right)-I_{c}\left(0,\omega_{s},\omega_{0}+\omega \right)+I_{c}\left(t_{f},\omega_{s},\omega_{0}-\omega \right)-I_{c}\left(0,\omega_{s},\omega_{0}-\omega \right)] \end{array}$$

with $\omega_{s}=\frac{\delta \omega }{2}$. The results achieved with this approach are described in Section 4.

## Appendix D. Application of LQOCT to ICR

We apply in this section the PMP to ICR processes in the case without any amplitude constraint. We denote by $X_{k}$ the state associated with the frequency $\omega_{k}$ as defined in Equation (3) of Section 2.1 and by $\left(X_{1}^{\left(k\right)},X_{2}^{\left(k\right)},X_{3}^{\left(k\right)}\right)$ the coordinates. $\left\{\omega_{k}\right\}$ is the set of discrete frequencies used in the numerical optimization. The optimal problem is defined through the cost functional J to minimize:

$$J=\frac{1}{2}\sum_{k}\left[{\left(X_{1}^{\left(k\right)}\left(t_{f}\right)-X_{1f}^{\left(k\right)}\right)}^{2}+{\left(X_{2}^{\left(k\right)}\left(t_{f}\right)-X_{2f}^{\left(k\right)}\right)}^{2}\right]+\frac{\lambda }{2}\int_{0}^{t_{f}}e_{x}^{2}dt.$$

Since there is no final condition on $X_{3}\left(t_{f}\right)$, this term does not appear in the expression of J. The Pontryagin Hamiltonian is given by:

$$H_{P}=\sum_{k}\left[p_{1}^{\left(k\right)}X_{2}^{\left(k\right)}-\omega_{k}^{2}p_{2}^{\left(k\right)}X_{3}^{\left(k\right)}+p_{3}^{\left(k\right)}X_{2}^{\left(k\right)}+p_{3}^{\left(k\right)}u\right]-\frac{\lambda }{2}e_{x}^{2}.$$

For the adjoint state, we have:

$$\left\{\begin{array}{c} {\dot{p}}_{1}^{\left(k\right)}=0\\ {\dot{p}}_{2}^{\left(k\right)}=-p_{1}^{\left(k\right)}-p_{3}^{\left(k\right)}\\ {\dot{p}}_{3}^{\left(k\right)}=\omega_{k}^{2}p_{2}^{\left(k\right)} \end{array}\right.$$

with the final conditions:

(A2) $$\left\{\begin{array}{c} p_{1}^{\left(k\right)}\left(t_{f}\right)=X_{1f}^{\left(k\right)}-X_{1}^{\left(k\right)}\left(t_{f}\right)\\ p_{2}^{\left(k\right)}\left(t_{f}\right)=X_{2f}^{\left(k\right)}-X_{2}^{\left(k\right)}\left(t_{f}\right)\\ p_{3}^{\left(k\right)}\left(t_{f}\right)=0 \end{array}\right.$$

Note that $p_{1}^{\left(k\right)}$ is a constant of the motion. We deduce the dynamics of the adjoint state:

(A3) $$\left\{\begin{array}{c} p_{1}^{\left(k\right)}\left(t\right)=p_{1}^{\left(k\right)}\left(t_{f}\right)\\ p_{2}^{\left(k\right)}\left(t\right)=A^{\left(k\right)}\cos\left(\omega_{k}t\right)+B^{\left(k\right)}\sin\left(\omega_{k}t\right)\\ p_{3}^{\left(k\right)}\left(t\right)=-p_{1}^{\left(k\right)}\left(t_{f}\right)+\omega_{k}\left[A^{\left(k\right)}\sin\left(\omega_{k}t\right)-B^{\left(k\right)}\cos\left(\omega_{k}t\right)\right] \end{array}\right.$$

with

$$\left\{\begin{array}{c} A^{\left(k\right)}=\sin\left(\omega_{k}t_{f}\right)\frac{p_{1}^{\left(k\right)}\left(t_{f}\right)}{\omega_{k}}+p_{2}^{\left(k\right)}\left(t_{f}\right)\cos\left(\omega_{k}t_{f}\right)\\ B^{\left(k\right)}=\sin\left(\omega_{k}t_{f}\right)p_{2}^{\left(k\right)}\left(t_{f}\right)-\frac{p_{1}^{\left(k\right)}\left(t_{f}\right)}{\omega_{k}}\cos\left(\omega_{k}t_{f}\right) \end{array}\right.$$

The optimal control $e_{x}^{*}$ can be expressed as:

(A4) $$e_{x}^{*}\left(t\right)=\frac{1}{\lambda }\sum_{k}p_{3}^{\left(k\right)}\left(t\right)$$

which can be transformed into:

$$e_{x}^{*}\left(t\right)=\frac{1}{\lambda }\sum_{k}\left[-p_{1}^{\left(k\right)}\left(t_{f}\right)+p_{1}^{\left(k\right)}\left(t_{f}\right)\cos\left(\omega_{k}\left(t_{f}-t\right)\right)-p_{2}^{\left(k\right)}\left(t_{f}\right)\omega_{k}\sin\left(\omega_{k}\left(t_{f}-t\right)\right)\right].$$

The last step consists in computing the trajectory corresponding to this optimal control field. We obtain for an ion of frequency ω:

$$\begin{array}{ccc} & & X_{1}\left(t_{f}\right)=\frac{1}{\lambda }\sum_{k}[p_{1}^{\left(k\right)}\left(t_{f}\right)\left(t_{f}-\frac{\sin(\omega t_{f})}{\omega }-\frac{\sin(\omega_{k}t_{f})}{\omega_{k}}\right)+p_{2}^{\left(k\right)}\left(t_{f}\right)(1-\cos\left(\omega_{k}t_{f}\right)\\ & & +\frac{p_{1}^{\left(k\right)}\left(t_{f}\right)}{2}\left[\frac{\sin(\left(\omega_{k}+\omega \right)t_{f})}{\omega_{k}+\omega }+\frac{\sin(\left(\omega_{k}-\omega \right)t_{f})}{\omega_{k}-\omega }\right]+\frac{\omega_{k}p_{2}^{\left(k\right)}\left(t_{f}\right)}{2}\left[\frac{\cos(\left(\omega_{k}+\omega \right)t_{f})-1}{\omega_{k}+\omega }+\frac{\cos(\left(\omega_{k}-\omega \right)t_{f})-1}{\omega_{k}-\omega }\right]] \end{array}$$

and

$$\begin{array}{ccc} & & X_{2}\left(t_{f}\right)=\frac{-\omega }{\lambda }\sum_{k}[p_{1}^{\left(k\right)}\left(t_{f}\right)\frac{\cos(\omega t_{f})-1}{\omega }+\frac{p_{1}^{\left(k\right)}\left(t_{f}\right)}{2}\left[\frac{1-\cos(\left(\omega_{k}+\omega \right)t_{f})}{\omega_{k}+\omega }+\frac{\cos(\left(\omega_{k}-\omega \right)t_{f})-1}{\omega_{k}-\omega }\right]\\ & & +\frac{\omega_{k}p_{2}^{\left(k\right)}\left(t_{f}\right)}{2}\left[\frac{\sin(\left(\omega_{k}+\omega \right)t_{f})}{\omega_{k}+\omega }-\frac{\sin(\left(\omega_{k}-\omega \right)t_{f})}{\omega_{k}-\omega }\right]] \end{array}$$

Such results can be written in a compact form as follows:

$$\left\{\begin{array}{c} \lambda X_{1}^{\left(j\right)}\left(t_{f}\right)=\sum_{k}\left[R_{jk}p_{1}^{\left(k\right)}\left(t_{f}\right)+S_{jk}p_{2}^{\left(k\right)}\left(t_{f}\right)\right]\\ \lambda X_{2}^{\left(j\right)}\left(t_{f}\right)=\sum_{k}\left[T_{jk}p_{1}^{\left(k\right)}\left(t_{f}\right)+U_{jk}p_{2}^{\left(k\right)}\left(t_{f}\right)\right] \end{array}\right.$$

where the matrices R, S, T and U are known explicitly and the index *j* labels the ion of the ensemble. We finally arrive at the following system to fulfill:

$$\left\{\begin{array}{c} \sum_{k}\left[R_{jk}X_{1f}^{\left(k\right)}+S_{jk}X_{2f}^{\left(k\right)}\right]=\lambda X_{1}^{\left(j\right)}\left(t_{f}\right)+\sum_{k}\left[R_{jk}X_{1}^{\left(k\right)}\left(t_{f}\right)+S_{jk}X_{2}^{\left(k\right)}\left(t_{f}\right)\right]\\ \sum_{k}\left[T_{jk}X_{1f}^{\left(k\right)}+U_{jk}X_{2f}^{\left(k\right)}\right]=\lambda X_{2}^{\left(j\right)}\left(t_{f}\right)+\sum_{k}\left[T_{jk}X_{1}^{\left(k\right)}\left(t_{f}\right)+U_{jk}X_{2}^{\left(k\right)}\left(t_{f}\right)\right] \end{array}\right.$$

In matrix form, for N=2, we have:

$$\left(\begin{array}{c} \sum_{k}R_{1k}X_{1f}^{\left(k\right)}+S_{1k}X_{2f}^{\left(k\right)}\\ \sum_{k}T_{1k}X_{1f}^{\left(k\right)}+U_{1k}X_{2f}^{\left(k\right)}\\ \sum_{k}R_{2k}X_{1f}^{\left(k\right)}+S_{2k}X_{2f}^{\left(k\right)}\\ \sum_{k}T_{2k}X_{1f}^{\left(k\right)}+U_{2k}X_{2f}^{\left(k\right)} \end{array}\right)=\left(\begin{array}{cccc} \lambda +R_{11} & S_{11} & R_{12} & S_{12}\\ T_{11} & \lambda +U_{11} & T_{12} & U_{12}\\ R_{21} & S_{21} & \lambda +R_{22} & S_{22}\\ T_{21} & U_{21} & T_{22} & \lambda +U_{22} \end{array}\right)\left(\begin{array}{c} X_{1}^{\left(1\right)}\left(t_{f}\right)\\ X_{2}^{\left(1\right)}\left(t_{f}\right)\\ X_{1}^{\left(2\right)}\left(t_{f}\right)\\ X_{2}^{\left(2\right)}\left(t_{f}\right) \end{array}\right)$$

This linear system allows computing the final state of the system $X_{k}\left(t_{f}\right)$, then the adjoint state from Equations (A2) and (A3) and the optimal control field with Equation (A4). We observe that the control law is expressed as a linear combination of cosine and sine functions of the frequencies $\omega_{k}$ of the finite discretized set. Numerical results are presented in Section 4. Note that the same method can be applied in the RWA starting from Equation (6) (see [50] for details).
